# *Heteroxiphia* Saini & Singh (Hymenoptera, Xiphydriidae), a genus new to China with descriptions of two new species

**DOI:** 10.3897/zookeys.102.860

**Published:** 2011-06-02

**Authors:** Meicai Wei, Gengyun Niu

**Affiliations:** College of Life Science and Technology, Central South University of Forestry and Technology, 498 South Shaoshan Road, Changsha 410004, P. R. China

**Keywords:** Hymenoptera, Xiphydriidae, *Heteroxiphia*, new species, China, India

## Abstract

*Heteroxiphia* Saini & Singh, 1987 is redescribed and *Heteroxiphia sinica* **sp. n.**and *Heteroxiphia tenuipalpa* **sp. n.** from China are described. A key to three species is provided and a key for separation of *Heteroxiphia*, *Trixiphidia* Wei, 1999 and *Yangixiphia* Wei, 2002 is also provided.

## Introduction

Specimens of Xiphydriidae are rarely collected in the field and many species are represented in collections by only one or a few specimens. Maa (1949) revised the Asiatic taxa of the family but *Heteroxiphia* and its type species, *Heteroxiphia maai* Saini & Singh, 1987 were subsequently described by Saini and Singh (1987) from northwestern India based on a single female. In 2000 and 2007, two specimens of Xiphydriidae were collected separately from Henan and Gansu Provinces, China. They represent two undescribed species of *Heteroxiphia*. The genus is redescribed based on new material and two new species are described below.

## Material and methods

Terminology of sawfly genitalia follows Ross (1945). Wing venation follows Niu and Wei (2010, Plate 1).

The images were obtained using a Nikon D2x digital camera and Motic BA400 microscope and further processed with Helicon Focus 5.1(©HeliconSoft) and Adobe Photoshop CS2 software.

Abbreviations used are: OOL = distance between the eye and outer edge of lateral ocellus; POL = distance between the mesal edges of the lateral ocelli; OCL = distance between a lateral ocellus and the occipital carina or hind margin of the head; and CD = the ratio of the distance between the cenchri and the breadth of a cenchrus.

Type specimens of the new species are deposited in the Insect Collection of Central South University of Forestry and Technology, Changsha, P. R. China.

## Taxonomy

### Heteroxiphia

Saini & Singh, 1987

http://species-id.net/wiki/Heteroxiphia

Heteroxiphia  Saini & Singh, 1987: 356. Type species: *Heteroxiphia maai* Saini & Singh, 1987, by original designation.

#### Description.

Small, body length 11–13 mm. Clypeus with an acute middle tooth; head almost as broad as thorax, not strongly extended behind eyes, lateral sides roundly narrowed in dorsal view; breadth of upper part of hind orbit distinctly longer than eye breadth but not much longer than long axis of eye; genal carina developed, extending to upper part of hind orbit; occipital carina almost complete, very narrowly separated at posterior margin of postocellar area; temple and postocellar area polished, very sparsely punctured; lower half of hind orbit with longitudinal carinae; distance between antennal sockets 2 times breadth of inner orbit and 2 times distance between antennal socket and anterior margin of clypeus; eyes short elliptical, inner margins indistinctly divergent downward in front view, distance between eyes at level of antennal sockets about 1.4–1.5 times height of eye; malar space (the entire distance from the eye to the lower edge of the antennal groove) distinctly longer than pedicel, about 1.5–2 times diameter of middle ocellus, with a large fovea; frons with curved and irregular carinae and punctures, supraclypeal area with regular longitudinal carinae; mandibles each with four teeth; maxillary palp with 3 palpomeres, first palpomere shortest, second palpomere slender and longest; labial palp with 3 palpomeres, first palpomere slender and longest, third palpomere more or less enlarged with an elliptical disc (sensory pit). Antenna shorter than head and thorax together, weakly compressed, strongly tapering toward apex, with 15–19 antennomeres, third antennomere shorter than 4th and 5th antennomeres together, each antennomere not broader than long. Anterior margin of pronotum deeply and broadly emarginated, middle part of pronotum very narrow; length of propleura in ventral view about 1.5 times as long as broad; mesoscutellum without tubercle, CD= 3.2–3.5; inner tibial spur of front leg bifurcate at apex, outer tibial spur minute; hind femur about 3.5–4 times longer than broad; apical tarsomeres not strongly enlarged; tarsal claws small, inner tooth of fore and middle claws slightly shorter than half length of outer tooth, hind claw with a very small inner tooth. Wings hyaline, forewing with vein 2r present, cell R1 broadly open at apex, anal cell with a cross vein at about apical 1/4; cells R1, Rs, M and A in hind wing closed. Body black with some white maculae.

#### Distribution.

China (Henan, Gansu); India (Himachal Pradesh).

#### Remarks.

Saini and Singh (1987, fig. 3) stated and figured that members of *Heteroxiphia* have four labial palpomeres. Observation of the labium of the two Chinese species shows that the basal short ring in Fig. 3 of Saini and Singh (1987) is an elevated platform of the labium, thus the labial palp has only three palpomeres.

*Heteroxiphia* is recognized by a combination of the following characters: maxillary palp with 3 palpomeres, the second palpomere much longer than the first and third palpomeres; labial palp also with 3 palpomeres; hind claw with a minute inner tooth; cell R1 in forewing broadly open, cell R1 in hind wing closed; face and lower half of hind orbit with regular longitudinal carinae; malar space about 1.5 times diameter of middle ocellus and with a large fovea; body black with some white maculae.

*Heteroxiphia* is closely allied to *Trixiphidia* Wei, 1999 (Wei and Xiao 1999). These are the only two genera of Xiphydriidae with three maxillary and labial palpomeres. *Yangixiphia* Wei, 2002 has also three maxillary palpomeres. The following key distinguishes the three genera.

**Table:** 

1	Cell R1 in forewing closed; labial palp with four palpomeres, the second palpomere longer than the third. China (Guizhou)	*Yangixiphia* Wei, 2002
–	Cell R1 in forewing open; labial palp with three palpomeres, the third palpomere longer than the second	2
2	Cell R1 in hind wing open; each claw with a long inner tooth close to and hardly shorter than outer tooth; face and hind orbits coarsely punctate, without regular carinae; maxillary palp with second palpomere about as long as third palpomere; labial palp with second palpomere more than 3 times as long as broad, third palpomere slender, hardly enlarged (Figs 1–2 in Wei and Xiao 1999). China (Henan)	*Trixiphidia* Wei, 1999
–	Cell R1 in hind wing closed; fore and middle claws each with a small inner tooth remote from and about 1/2–1/3 length of outer tooth, hind claw with a minute inner tooth; face and hind orbits with regular carinae, not punctate; maxillary palp (Figs 5, 13) with second palpomere much longer than third palpomere; labial palp (Figs 4, 12) with second palpomere about 1.5–2 times as long as broad, third palpomere short and distinctly enlarged. China (Henan, Gansu); India (Himachal Pradesh)	*Heteroxiphia* Saini & Singh, 1987

#### Heteroxiphia sinica

Wei & Niu sp. n.

urn:lsid:zoobank.org:act:15136759-A469-4BEB-8632-2CE1B705E3BB

http://species-id.net/wiki/Heteroxiphia_sinica

Figs 1–9 

##### Description. Female

(holotype, Fig. 8). Body length 11mm. Black, a long and broad stripe on inner orbit, a short stripe on lateral corner of clypeus, a large X-shaped mark on face and anterior margin of frons, malar space (Fig. 2), a broad and long stripe on hind orbit (Fig. 3), outer margin and posterior corner of pronotum, tegula, an elliptical spot on posterior part of lateral lobe of mesoscutum, a round mark on lateral side of mesoscutellum, cenchrus, lateral mark on metascutellum, a strongly curved and narrow middle stripe on first abdominal tergite, a broad transverse band on second abdominal tergite, a short band on third abdominal tergite, a minute lateral dot on 4th abdominal tergite and a long band on 8th abdominal tergite (Figs 7, 8), white; legs black, each tibia and tarsus white, 4th tarsomeres and apical half of each terminal tarsomere dark brown. Body hairs silver. Wings hyaline, stigma and veins dark brown.

**Figures 1–9:**
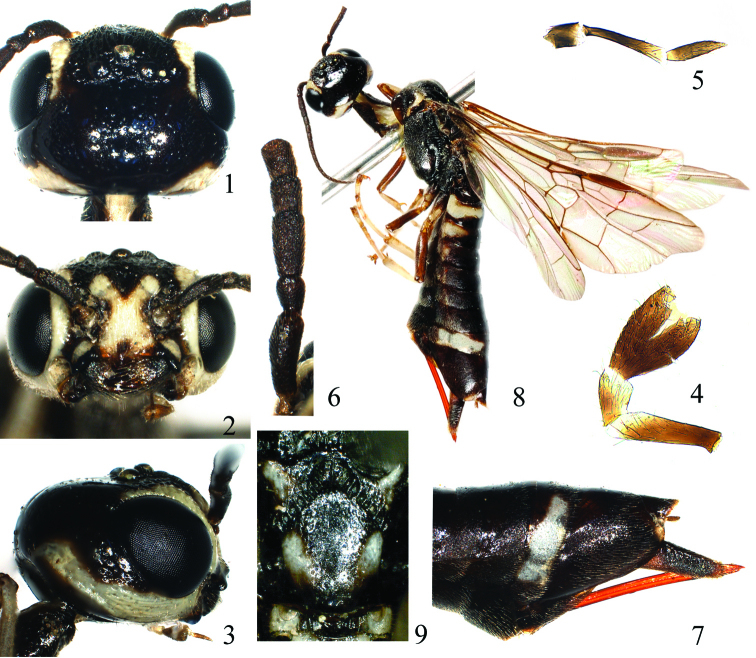
*Heteroxiphia sinica* sp. n., holotype **1** Head, dorsal view **2** Head, front view **3** Head, lateral view **4** Labial palp **5** Maxillary palp **6** 1st–5th antennomeres **7** Apex of abdomen, lateral view **8** Adult female, lateral view **9** Mesoscutellum

Clypeus, face and frons with distinct longitudinal carinae and microsculpture, lateral part of frons densely punctured; vertex and upper part of hind orbit sparsely punctured; head behind eyes strongly shiny (Fig. 1); dorsal side of pronotum densely punctured, lateral lobe largely polished, shiny, bottom of furrows with a row of short carinae; dorsal side of propleuron shiny with some large punctures, ventral side of propleuron densely punctured and microsculptured; mesonotum minutely and densely punctured, lateral sides and posterior half of mesoscutellum sparsely punctured, shiny; bottom of furrows on mesonotum with a row of short carinae; metascutellum densely punctured; mesopleuron and metapleuron coarsely and densely punctured, mat, lower posterior corner glossy, impunctate; first abdominal tergite sparsely punctured, shiny; second abdominal tergite glossy, lateral side with some punctures, basal 2/3–4/5 of other tergites densely microsculptured, weakly shiny; abdominal sternites microsculptured with obscure punctures, feebly shiny; basal sheath polished, apical sheath microsculptured.

Distance between eyes at clypeus level about 1.4 times eye height; malar space 1.3 times length of pedicel (Fig. 2); middle fovea furrow like, broad, lateral fovea punctiform; face and front distinctly above top of eyes (Figs 2, 3); interocellar furrow obscure, postocellar furrow fine, curved; POL: OOL: OCL = 5: 8: 18; vertex roundly convex (Figs 1, 3); lateral side of temple shorter than eye in dorsal view (Fig. 1); occipital carina and genal carina developed, close to each other near lateral corner of postocellar area; length ratio of maxillary palpomeres about 3: 7: 5, first palpomere short, slightly longer than broad, second palpomere 6 times longer than broad, distinctly broadened toward apex, third palpomere 4.3 times longer than broad, apical part strongly tapering (Fig. 5); labial palp with 3 palpomere, first palpomere slightly (1.05×) longer than third palpomere, third palpomere strongly enlarged, 2 times as long as second palpomere (Fig. 4). Antenna with 19 antennomeres, slightly shorter than 2 times head breadth, basal part of flagellum weakly compressed, strongly tapering toward apex (Fig. 8), length ratio of basal 5 antennomeres: 18: 7: 13: 7: 7; hairs on antennomeres quite procumbent (Fig. 6). Mesoscutellum 1.25 times as long as broad, distinctly narrowed backward and strongly protruding forward (Fig. 9); cenchrus small, CD=3.5; central part of metascutellum concave. Inner tibial spur of fore leg bifurcate at apex; metabasitarsus slightly shorter than following 4 tarsomeres together; fore and middle claw with inner tooth slightly shorter than half length of outer tooth, inner tooth of hind claw slightly shorter than 1/3 length of outer tooth. Vein Sc in forewing distinctly basal to Rs, 2r curved and interstitial to 1r-m, cell 2Rs slightly shorter than 1Rs, cell 1M about 1.8 times longer than broad, first abscissa of Rs slightly longer than first abscissa of vein 1M, cu-a 1.5 times length of and interstitial to first abscissa of vein 1M; cell R1 in hind wing with a short apical stump, cell M as long as Rs, apex of anal cell acute, upper part of cu-a distinctly oblique inwards. Ovipositor sheath (distance between base of basal sheath and apex of apical sheath) about as long as hind tibia and metabasitarsus together, strongly bent ventrally (Fig. 7), apical sheath about 4 times longer than broad in dorsal view.

##### Male.

Unknown.

##### Distribution.

China (Henan Province).

##### Etymology.

This is the first Chinese species of the genus and so it is named as *sinica*.

##### Holotype

♀, China: Jiyuan, Huanglianshu, 1700 m, 2000.VI.7, Wei Meicai leg.

##### Remarks.

See the key to species for differences between *Heteroxiphia sinica* and other two species of the genus.

#### Heteroxiphia tenuipalpa

Wei & Niu sp. n.

urn:lsid:zoobank.org:act:A50ADBE5-4F03-45B6-9D31-1AC2A2D94227

http://species-id.net/wiki/Heteroxiphia_tenuipalpa

Figs 10–18 

##### Description. Female

**Figures 10–18:**
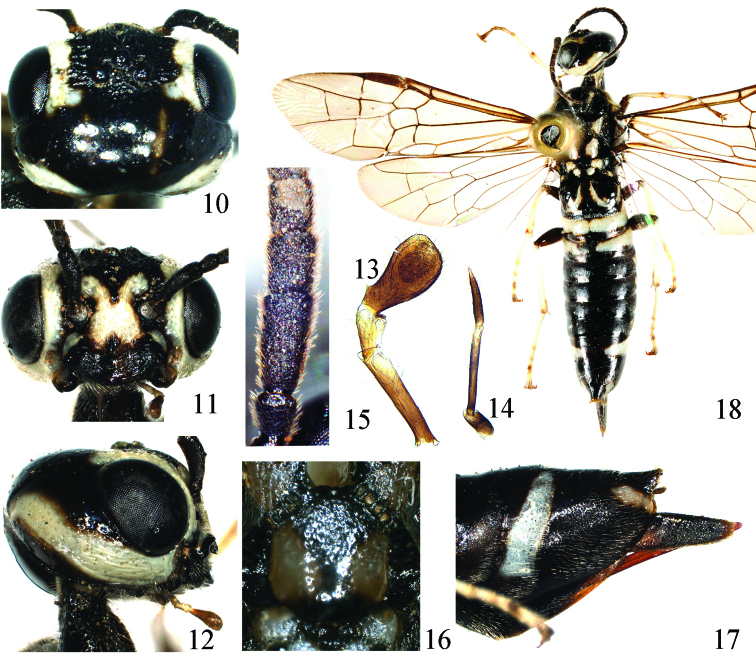
*Heteroxiphia tenuipalpa* sp. n., holotype **10** Head, dorsal view **11** Head, front view **12** Head, lateral view **13** Labial palp **14** Maxillary palp **15** 1st–4th antennomeres **16** Mesoscutellum **17** Apex of abdomen, lateral view **18** Adult female, dorsal view

(holotype, Fig. 18). Body length 13 mm. Black, a long and broad stripe on inner orbit, a short stripe on lateral corner of clypeus, a large X-shaped mark on face and anterior margin of frons, malar space (Fig. 11), a broad and long stripe on hind orbit (Fig. 12), narrow anterior margin, broad lateral and posterior margins of pronotum, tegula, lateral stripe on prescutum, an elliptical spot on posterior part of lateral lobe of mesoscutum, a round mark on lateral side of mesoscutellum, cenchrus, lateral mark on metascutellum, a strongly curved middle stripe on first abdominal tergite, a broad transverse band on second abdominal tergite, a medially separated band on third abdominal tergite (Fig. 18), a small lateral spot on 4th and 5th abdominal tergites, a long band on 8th abdominal tergite and a short stripe on posterior corner of 9th tergite (Fig. 17), white; lateral side of postocellar area with obscure brown stripe (Fig. 10); legs black, apex of hind coxa and hind trochanter brown, each tibia and tarsus white, extreme apex of hind tibia, tibial spurs, 4th tarsomere and apical half of each terminal tarsomere black brown. Body hairs silver. Wings hyaline, apical part slightly infuscate, stigma and veins dark brown.

Clypeus, face and frons with distinct longitudinal carinae and microsculpture, lateral part of frons densely punctured; vertex and upper part of hind orbit sparsely punctured, head behind eyes strongly shiny (Fig. 10); dorsal side of pronotum densely punctured, lateral lobe largely polished, shiny, bottom of middle and lateral furrows with short carinae; dorsal side of propleuron shiny with some large punctures, ventral side of propleuron densely punctured and microsculptured; mesonotum minutely and densely punctured, lateral sides and posterior 2/3 of mesoscutellum sparsely punctured, shiny; bottom of furrows on mesonotum with many short carinae; metascutellum coarsely punctured; mesopleuron and metapleuron coarsely and densely punctured, mat, lower posterior corner glossy, impunctate; first abdominal tergite sparsely punctured, shiny; second abdominal tergite glossy, lateral side with some punctures, basal 4/5 of other tergites densely microsculptured, weakly shiny; abdominal sternites microsculptured with obscure punctures, feebly shiny; basal sheath polished, apical sheath microsculptured.

Distance between eyes at clypeus level about 1.5 times eye height; malar space 1.3 times length of pedicel (Fig. 11); middle fovea round, lateral fovea punctiform; face and front distinctly above top of eyes (Figs 11, 12); interocellar furrow obscure, postocellar furrow fine, curved; POL: OOL: OCL = 5: 9: 20; vertex roundly convex (Figs 10, 12); lateral side of temple slightly longer than eye in dorsal view; occipital carina and genal carina developed, close to each other near lateral corner of postocellar area; maxillary palp slender, length ratio of palpomeres about 3: 7: 5, first palpomere short, 2 times longer than broad, second palpomere 10 times longer than broad, not broadened toward apex, third palpomere 7 times longer than broad, gradually tapering toward apex (Fig. 14); labial palp with 3 palpomeres, first palpomere 1.25 times length of third palpomere, third palpomere strongly enlarged, 2 times as long as second palpomere (Fig. 13). Antenna with 19 antennomeres, slightly shorter than 2 times head breadth, basal part of flagellum weakly compressed, strongly tapering toward apex (Fig. 18), length ratio of basal 5 antennomeres: 18: 7: 15: 9: 8; hairs on antennomeres oblique, not procumbent (Fig. 15). Mesoscutellum about as long as broad, not narrowed posteriorly and roundly protruding anteriorly (Fig. 16); cenchrus small, CD=3.2; central part of metascutellum concave. Inner tibial spur of fore leg bifurcate at apex; hind basitarsus slightly shorter than following 4 tarsomeres together (10: 11); fore and middle claws with inner tooth slightly shorter than half length of outer tooth, inner tooth of hind claw about 1/3 length of outer tooth. Vein Sc in forewing interstitial with base of vein Rs, 2r curved and interstitial with 1r-m, cell 2Rs slightly shorter than 1Rs, cell 1M about 1.8 times longer than broad, first abscissa of Rs as long as first abscissa of vein 1M, cu-a 1.5 times length of and interstitial to first abscissa of vein 1M; cell R1 in hind wing with a short apical stump, cell M as long as Rs, apex of anal cell acute, upper part of cu-a distinctly oblique inwards. Ovipositor sheath 1.2 times as long as hind tibia and metabasitarsus together, distinctly bent ventrally (Fig. 17), apical sheath slightly more than 4 times longer than broad in dorsal view.

##### Male.

Unknown.

##### Distribution.

China (Gansu Province).

##### Etymology.

This species is named after its slender maxillary palp.

##### Holotype

♀, China: Gansu, Maiji, Dongcha Forest Plant, 2007.VI.13, Wu Xingyu leg.

##### Remarks.

See the following key to species for differences between *Heteroxiphia sinica* and other two species of the genus.

### Key to species of Heteroxiphia

**Table:** 

1	Mandible and mesopleuron with distinct white maculae; upper half of inner orbit black, without white stripe; frons coarsely punctured without regular carinae; inner tooth of hind claw minute, shorter than 1/4 length of outer tooth; vein cu-a in forewing distinctly apical to base of vein 1M; mesoscutellum densely punctured; third maxillary palpomere narrower than second palpomere; antenna with 15 antennomeres. India: Himachal Pradesh	*Heteroxiphia maai*
–	Mandible and mesopleuron black, without distinct white maculae; entire inner orbit with broad white stripe; frons with regular carinae; inner tooth of hind claw distinct, about 1/3 length of outer tooth; vein cu-a in forewing interstitial with base of vein 1M; mesoscutellum sparsely punctured in posterior half; third maxillary palpomere stouter than second palpomere; antenna with 19 antennomeres. China	**2**
2	Prescutum entirely black; mesoscutellum longer than broad, narrowed posteriorly (Fig. 9); maxillary palp shorter and stouter, second palpomere 6 times longer than broad, distinctly broadened toward apex, third palpomere 4.3 times longer than broad (Fig. 5); temple shorter than eye in dorsal view (Fig. 1); hairs on antennomeres procumbent (Fig. 6); first palpomere of labial palp 1.05 times longer than third palpomere; vein Sc in forewing distinctly basal to base of vein Rs; ovipositor sheath as long as hind tibia and hind basitarsus together. China: Henan	*Heteroxiphia sinica*
–	Lateral side of prescutum white; mesoscutellum as long as broad, not narrowed posteriorly (Fig. 16); maxillary palp very slender, second palpomere 10 times longer than broad, not broadened toward apex, third palpomere 7 times longer than broad (Fig. 14); temple longer than eye in dorsal view; hairs on antennomeres oblique, not procumbent (Fig. 15); first palpomere of labial palp 1.25 times longer than third palpomere; vein Sc in forewing interstitial with base of vein Rs; ovipositor sheath 1.2 times as long as hind tibia and hind basitarsus together. China: Gansu	*Heteroxiphia tenuipalpa*

## Supplementary Material

XML Treatment for Heteroxiphia
